# Practical Guidelines: Lung Transplantation in Patients with Cystic Fibrosis

**DOI:** 10.1155/2014/621342

**Published:** 2014-03-30

**Authors:** T. O. Hirche, C. Knoop, H. Hebestreit, D. Shimmin, A. Solé, J. S. Elborn, H. Ellemunter, P. Aurora, M. Hogardt, T. O. F. Wagner, ECORN-CF Study Group

**Affiliations:** ^1^Department of Pulmonary Medicine, German Clinic for Diagnostics (DKD), Wiesbaden, Germany; ^2^Department of Chest Medicine, Erasme University Hospital, Brussels, Belgium; ^3^Department of Pediatrics, University Hospital Wuerzburg, Germany; ^4^Regional Adult CF Centre, Belfast City Hospital, Belfast, UK; ^5^Lung Transplant Unit, Hospital Universitario La Fe, Valencia, Spain; ^6^Centre for Infection and Immunity, Queens University, Belfast and Adult CF Centre BHSCT, UK; ^7^Cystic Fibrosis Center, Department of Pediatrics, Medical University Innsbruck, Innsbruck, Austria; ^8^Paediatric Respiratory Medicine and Lung Transplantation, Great Ormond Street Hospital, London, UK; ^9^Institute for Medical Microbiology and Infection Control, Goethe University Hospital Frankfurt, Germany; ^10^Department of Pneumology, Goethe University Hospital Frankfurt, Germany; ^11^Department of Pneumology, Frankfurt University Hospital, Building 15B,Theodor-Stern-Kai 7, 60590 Frankfurt, Germany

## Abstract

There are no European recommendations on issues specifically related to lung transplantation (LTX) in cystic fibrosis (CF). The main goal of this paper is to provide CF care team members with clinically relevant CF-specific information on all aspects of LTX, highlighting areas of consensus and controversy throughout Europe. Bilateral lung transplantation has been shown to be an important therapeutic option for end-stage CF pulmonary disease. Transplant function and patient survival after transplantation are better than in most other indications for this procedure. Attention though has to be paid to pretransplant morbidity, time for referral, evaluation, indication, and contraindication in children and in adults. This review makes extensive use of specific evidence in the field of lung transplantation in CF patients and addresses all issues of practical importance. The requirements of pre-, peri-, and postoperative management are discussed in detail including bridging to transplant and postoperative complications, immune suppression, chronic allograft dysfunction, infection, and malignancies being the most important. Among the contributors to this guiding information are 19 members of the ECORN-CF project and other experts. The document is endorsed by the European Cystic Fibrosis Society and sponsored by the Christiane Herzog Foundation.

## 1. Introduction

All aspects of CF care have been optimised over recent decades and survival continues to progressively improve; end-stage respiratory insufficiency at an adult age remains the cause of death in the vast majority of CF patients. CF constitutes the third major indication for lung transplantation (LTX) after emphysema and pulmonary fibrosis. LTX has the potential to significantly extend survival and improve quality of life (QoL) provided that candidates are referred on time to the transplant centre and carefully selected. Since CF is a multiorgan disease, various particularities and CF-specific complications must be taken into consideration before and after LTX, which requires a close cooperation between CF paediatricians/pulmonologists and the transplant team.

This paper was initiated in March 2012 in Obergurgl/Austria during a workshop on LTX in CF. A first draft in German was compiled based on the experts' workshop presentations and was subsequently translated into English. This English version was presented to a wider audience at an ECORN-CF (ECORN-CF: European Centres of Reference Networks for Cystic Fibrosis (ecorn-cf.eu)) meeting during the ECFS conference in Dublin/Ireland in June 2012 and working groups were established to subsequently develop the various subsections. This revision was done according to a modified (simplified to two iterations) Delphi procedure among all participants. A final revision meeting took place in April 2013 in Frankfurt/Germany.

The paper was compiled to present the latest developments in science and technology in the field of LTX for CF with particular emphasis on candidate selection, surgical preparation, and long-term care. It is intended to serve CF care team members as a guide and assist them in counselling CF patients and their families on all aspects of LTX. Since this paper was prepared by a European working group, some information given is more Europe specific and might not apply to other areas.

Questions on issues that are not included in this paper due to a lack of scientific references may be asked online at http://www.ecorn-cf.eu/.

## 2. Epidemiology of LTX for CF

Approximately 3700 lung transplantations are recorded worldwide per year by the International Society for Heart and Lung Transplantation (ISHLT) [1]. Since ISHLT registry is voluntary, the actual number of transplantations is assumed to be higher. Detailed data on waiting lists, number of transplantations, and mortality are available (http://www.ishlt.org/).

The main indications for bilateral LTX are at present emphysema (27%), CF (26%), and idiopathic pulmonary fibrosis (17%). For various reasons the relative proportion of CF LTX recipients is higher in Europe as compared to the United States (US) [1].

In the cohort of all LTX recipients transplanted worldwide from 1994 to 2010, the median actuarial survival after LTX was 6.7 years; for patients, who had survived the first year, the median actuarial survival increased to 9.4 years. For CF LTX recipients these median actuarial survival times were 7.5 and 10.4 years, respectively [1].

## 3. Preparation for LTX

Please note that the preparation procedures vary from country to country.

### 3.1. Formal Referral to the Transplant Centre

Given the shortage of organs, the resulting waiting times, and the unpredictable evolution of end-stage CF, CF patients eligible for LTX should be referred to a transplant centre at an appropriate time. An FEV_1_ < 30% of predicted values and/or a rapid decline in FEV_1_ despite optimal conservative treatment, malnutrition, and diabetes, female gender, frequent exacerbations and/or an increasing need for intravenous antibiotherapy, recurrent, massive hemoptysis, which cannot be controlled by bronchial artery embolisation, relapsing or complicated pneumothorax, or the need for ICU admission are all indicators that a pretransplant assessment is warranted [2, 3]. The patient's individual motivation, current QoL, and social environment need to be taken into consideration as well [2, 4, 5].

The patient must be fully informed about the long-term medical, psychological, and social effects of the anticipated transplantation. His explicit request for LTX, documented by written consent, is a fundamental prerequisite before being listed in some countries. Children and adolescents may not be listed against their will. Guidelines pertaining to this issue have been released in some countries [6].

### 3.2. Determination of the Suitable Time for Listing

Presentation at the transplant centre and admission to the waiting list are two separate decision steps. Pretransplant assessment should be carried out when there are indicators of a negative evolution but does not necessarily result in immediate listing. Acceptance onto the waiting list becomes necessary when LTX is likely to prolong life and improve QoL when compared to conservative medical treatment [7, 8]. Listing should happen when estimated life expectancy is reduced, but also at a time when it is still superior to the expected waiting time [2]. Expected waiting time may vary widely according to country, blood group, and height and these parameters should be taken into account. Still, it remains very difficult to reliably assess prognosis for an individual patient. Statistical and mathematical approaches with multivariate models for survival in the CF have not been able to identify robust predictors [3]. Country specific allocation systems play a crucial role in the decision when to list.

During the waiting period, the patient should pursue an active lifestyle in order to maintain muscle and bone mass and at least stabilise his nutritional condition. Vaccinations should be completed or boosted as needed. The social support network for the perioperative and postoperative phase should be strengthened and emotional preparation for the transplantation should be accomplished with professional help. At the same time continuous reevaluation of the medical situation is essential to make sure that any deterioration of health is detected and treated early on and results in a potential adaptation of the waiting list position (see below) [4].

### 3.3. Allocation of Donor Lungs

Eligible patients are put on the waiting list at their local transplant centre. In most European countries, they are then notified to an organ procurement organisation which assigns available donor lungs according to predetermined criteria. The process of organ distribution is called “organ allocation.” Allocation criteria may be based on geography (regional, national, and international), urgency (e.g., by audit process, individual decision, or objectively by a score system) or on waiting time, or a combination of several criteria.

*Eurotransplant* (http://www.eurotransplant.org/) is the largest organ procurement organisation in Europe, comprising Austria, Belgium, Croatia, Germany, Luxembourg, The Netherlands, Slovenia, and—from July 2013 onwards—Hungary. Spain and Portugal have their own network, as do the Scandinavian countries, France, and the UK.

Historically, lung allocation, in the US and the Eurotransplant region, was mainly based on waiting time. Recently, though, in the context of a high mortality of patients waiting for LTX (mainly of patients affected by pulmonary arterial hypertension and pulmonary fibrosis), a lung allocation score (LAS) has been devised [9]. The LAS takes into account the estimated survival benefit offered by LTX by 1 year after surgery and medical urgency. Since its introduction as a tool for donor lung allocation in the US in 2005, the number of LTX for US CF patients has increased by ~25%. Of note, 70% of wait-listed CF patients were transplanted after a waiting period of 1 year decreasing the 1-year waiting-list mortality from 15% to 10% [10]. The LAS system has also been recently adopted in Germany for LTX candidates age 12 and older. In the rest of the Eurotransplant region donor lung allocation is currently still based on waiting time and/or patient status. When waiting time is used, listing should be early.

### 3.4. Preoperative Examinations

An extensive work-up is necessary in preparation for LTX.  Table 1 shows the common prerequisites and others that may be necessary according to the individual circumstances (e.g., right heart catheterisation) [2, 4, 11]. The results of this work-up may also help improve the baseline condition prior to LTX. Examples would be an improved nutritional status, better physical fitness through physiotherapy and rehabilitation, or the treatment of a potential focus of infection (e.g., paranasal sinuses, teeth).

### 3.5. Impact of Pretransplant Upper Airways Colonisation on LTX Outcomes

Colonisation of the transplanted lung with pathogens such as* Pseudomonas aeruginosa (P.a.) *may damage the allograft both directly and indirectly and probably constitutes a risk factor for chronic lung allograft dysfunction (CLAD) (see below) [12]. Pathogens cross colonise between upper and lower airways in CF [13, 14] and strains recovered from the transplant may be genotypically identical to those colonising the upper airways and paranasal sinuses [14, 15]. It is not clear whether conservation and/or surgical methods may prevent graft recolonisation. Surgical approaches have been advocated in order to eradicate paranasal sinus infection either before or immediately after LTX. However, since the mucous membranes of the paranasal sinuses also show the CFTR gene defect, the underlying problem of defective mucociliary clearance cannot be surgically eliminated and in any case upper airway colonisation cannot be surgically eliminated either [16]. To date, there are no reports to indicate that survival rates of CF LTX recipients with and without prophylactic pre- or posttransplant sinus surgery differ [17]. Additional studies are required to evaluate the impact of surgical and/or conservative elimination of sinus colonisation on the long-term outcome after LTX.

### 3.6. General Absolute and Relative Contraindications to LTX

For each eligible patient, that is, who meets the criteria for LTX, potential contraindications should be discussed on a case by case basis with the transplant team, but not* per se* impede referral. Contraindications may evolve or become apparent during the waiting time and so eligibility should be regularly reconsidered.

Malignant diseases during the past 2 years are considered an absolute contraindication to LTX [2, 8], with the exception of nonmelanoma skin tumours such as squamous-cell and basal-cell carcinomas. Most transplant centres demand a disease-free interval of 5 years. Serious extrapulmonary diseases are another absolute contraindication when combined transplantations/surgical correction is impossible. These include severe chronic renal failure, severe hepatic failure, and coronary artery disease that cannot be optimised by interventional and surgical procedures or is associated with a significantly reduced left ventricular pump function. Furthermore, active infections, including active untreated tuberculosis and chronic active hepatitis B, are absolute contraindications. Hepatitis C is only a contraindication when it is active and results in significant histological hepatic damage [18, 19]. Many transplant centres include HIV infection in the list of absolute contraindications although successful transplantations in this setting have been carried out [20, 21].

Severe neuromuscular diseases as well as serious deformations of the spinal column and chest wall and advanced osteoporosis with fractures may constitute another group of absolute contraindications. However, scoliosis, a frequent finding in CF, is not a contraindication [22].

Finally, previously documented failure to adhere to medical therapies or to follow-up, untreatable mental illness, and acute or recent addictions (over the past 6 months) such as considerable tobacco, alcohol, and substance abuse (Table 2(a)) are absolute contraindications as well.

Malnutrition results in shorter survival after LTX in general [23], but there is no evidence that nutritional status does influence posttransplantation survival in CF patients [3, 24].

Systemic steroid therapy should be tapered to <20 mg prednisolone equivalent per day before transplantation. Pretransplant endotracheal intubation and mechanical ventilation can no longer be considered as contraindication, since transplantation in mechanically ventilated patients with CF is not associated with an increase in morbidity or mortality [25, 26]. In fact, invasive mechanical ventilation or extracorporeal membrane oxygenation (ECMO) is more and more often used as a bridge to LTX. Hemodynamic instability is at least a relative contraindication. Other illnesses such as diabetes mellitus, arterial hypertension, gastroesophageal reflux disease (GERD), and osteoporosis [24] will need optimal management prior to transplantation. A significantly reduced functional status without potential for rehabilitation is also regarded as a relative contraindication.

Colonisation with multi- or panresistant* P.a.* in CF patients does not result in inferior outcomes [27, 28]. Colonisation with MRSA, multi- or panresistant Gram-negative rods such as* Stenotrophomonas maltophilia* or* Achromobacter* species (xylosoxidans) is also no reason to deny LTX [28]. In contrast,* Burkholderia cepacia *complex (Bcc) infection in CF patients may be associated with septic clinical courses including the so-called “cepacia syndrome” when* B. cenocepacia*,* B. multivorans, *and* B. dolosa* are involved. The literature reports higher mortality rates after LTX for CF patients colonised with Bcc, especially for those colonised with* B. cenocepacia* and in some reports for those colonised with* B. multivorans* [29–32]. Dismal outcomes have also been reported in patients colonised with a Gram-negative bacteria closely related to Bcc,* Burkholderia gladioli* [29–33]. Many LTX centres therefore decline patients presenting with these pathogens (Table 2(b)). However, a case by case evaluation is mandatory [34].

Nontuberculous mycobacteria (NTM) also pose significant challenges after LTX (see below). The prevalence of NTM in CF patients ranges from 3 to 19% [35].* M. avium *complex (belonging to slowly growing mycobacteria) is most common in the US, whereas* M. abscessus *complex (belonging to rapidly growing mycobacteria) prevails in Europe. Detection of NTM is currently not rated as an absolute contraindication to LTX. Centers that accept patients with* M. abscessus* infection for transplantation usually advocate aggressive treatment prior to and after LTX [36].

### 3.7. Preoperative Nutritional Management

Lung function and nutrition are codependent variables in CF. Moreover, the nutritional status has a considerable impact on muscular function. Poor nutritional status is an independent risk factor for poor survival in CF [37–39].

Extremes of body weight, body mass index (BMI) and lean body mass (LBM) have been shown to negatively affect LTX outcome [24, 40–43]. A healthy weight should be promoted long before LTX is indicated [40]. This being said, CF patients with end-stage lung disease are at risk of significant weight loss as energy expenditure is increased on one hand (secondary to the increased work of breathing and the chronic bronchopulmonary infection) and optimal calorie uptake is compromised on the other hand (by the exocrine pancreatic insufficiency, a loss of appetite in the context of inflammation/infection, and the respiratory discomfort caused by gastric distension after meals) [44–46]. Relevant nutritional information should be exchanged between dietitians from the referring CF centre and the transplant centre to optimise management. Wait listing should not be unduly delayed while attempts are made to correct a low BMI, as this might result in a higher wait list mortality [9].

### 3.8. Psychosocial Evaluation and Support

#### 3.8.1. Psychosocial Evaluation

The psychosocial functional level of adolescent CF patients is compared to that of a normal random sample—*until the disease is taking a more serious course* [47]. In adult CF patients the prevalence of anxiety is higher whereas the prevalence of depression is lower when compared to the general population [48]. The emotional strain is even higher for relatives, with a third to half of the people interviewed being affected. The pretransplantation anxiety scores are nevertheless lower in CF patients, as they have better social support and more functional coping strategies, than in patients, who need LTX for other indications [49, 50].

Data on the significance of mental illnesses and on psychosocial indicators regarding the outcome of LTX are contradictory. Some authors suggest that mental illness is a relative or absolute contraindication [51], others emphasise that these disorders are relatively well treatable [52] or request suitable support services at transplantation centres [53]. Evaluation of LTX candidates by an external psychiatrist is unnecessary; instead it has been advocated to involve a psychologist, a psychiatrist, a psychosomatically experienced physician, and/or a social worker in the care-giving team [54]. If the CF and TX centre have different locations and teams, it is important to develop a good communication between them in order to enable the patient's psychosocial evaluation, support, and integration in the new team.

There are several instruments to measure adherence and psychosocial adjustment. An example would be the compact Transplant Evaluation Rating Scale (TERS) [55], which allows for a structured psychosocial evaluation and enables the examiner to assess the patient with regard to the following domains: substance abuse, compliance, health consciousness, social support, past coping, dealing with the current disease, affect and mental status.

Sex and age have an additional bearing on treatment adherence. Young female CF patients encounter significantly more emotional tension and concern about their disease, their degree of self-esteem is lower, they tend to be more readily disappointed about treatment, and their adherence in certain areas of treatment is inferior [56]. CF adolescents feel and behave differently from CF adults. The tasks of developing autonomy and shaping identity are radically changed when the indication of an organ transplantation is given [54]. The relationship with the doctor and the CF team is considered an important factor to promote adherence in CF patients [57].

#### 3.8.2. Psychosocial Counselling, Therapeutic Education, and Support Groups

Different forms of psychosocial support intend to enhance adherence, health-related QoL, the individual coping with illness-related anxiety, and the transplantation experience. While therapeutic education promotes active, cognitive, knowledge-based coping styles, psychosocial counselling and support groups focus more on emotional adaptation and integrating processes. Dealing with regression, the transplantation experience, or a postoperative delirium, it is also important to consider the patient's defence mechanisms and use it during post-TX counselling. The patient's mental representation of the donor and the integration of the transplanted organ are seen as a positive assimilation strategy or a disintegrative process [58].

Central topics in the counselling of CF patients prior to LTX include patient education, enhancement of self-efficiency, coping, and the definition of treatment objectives and purposes in life [54]. In the postoperative period, the desired extent of psychosocial counselling and support remains nearly as high as pre-TX. The prevalence of anxiety disorders is unchanged whereas depression decreases [53]. Rejection, side effects of medication, return to “normalcy,” feelings of guilt [59], and social and legal questions are among the primary issues for which psychosocial support and counselling are sought. Adolescent CF LTX recipients are also dealing with long-term goals (e.g., 80% wish to get married and have children), interpersonal relations (peers, feeling of belonging), and the desire to regain control over their everyday life and their illness. Psychological help and support for LTX patients have proved successful and have been firmly established in a number of transplant centres [60]. In addition, self-help organisations are increasingly taking over important tasks of counselling and support.

## 4. Perioperative Approach

### 4.1. Bridge to Transplant

At the preterminal stage of respiratory failure, extended intensive care measures may become necessary to stabilise the patient in imminently life-threatening situations. In the event of terminal failure of gas exchange, various temporary organ replacement options are available as “lung replacement” after conventional ventilation modalities have been exhausted. These extracorporeal life support systems (ECLS) enable direct oxygenation of or CO_2_ extraction from the blood. When LTX is indicated, these technologies (in particular* interventional lung assist (iLA), venovenous or venoarterial extracorporeal membrane oxygenation (VV-/VA-ECMO)*) may be used as a bridge to transplant. Experienced centres report 1-year survivals of 60–92% [61–63]. These bridging modalities are resource-intensive; they may be maintained for a limited time (a maximum of some/several weeks). Prompt organ availability is a prerequisite for a successful outcome in these situations.

### 4.2. Surgical Procedures

The surgical techniques for LTX are nowadays similar throughout the world. Therefore, we will not discuss well-known surgical details but will focus on special considerations in CF patients.

#### 4.2.1. Lung Donation and Allocation

While the vast majority of donor lungs are retrieved from donors after brain death (DBD), donation after circulatory death (DCD) has led to a substantial increase in available donor organs over the last years [64, 65]. Lungs from DCD donors have been successfully transplanted in CF patients. Furthermore,* ex vivo* lung perfusion technique (EVLP) is an emergent new option to increase the donor pool. It allows to evaluate otherwise unacceptable donor lungs over several hours after retrieval and to recondition them for subsequent LTX [66–68].

Donor lung allocation is principally based on blood group compatibility and size matching. Size matching is primarily based on donor and recipient predicted total lung capacity (TLC). It has to be taken into account that in CF recipients sometimes the real TLC can differ significantly from the predicted TLC [69]. At present, there is no matching for major histocompatibility antigens nor for viral (CMV, EBV) status of donor and recipient.

#### 4.2.2. Type of Lung Replacement

Given that a remaining CF lung would serve as a source of infection, CF patients generally require a bilateral LTX. The most widespread operative technique today is bilateral sequential LTX [70]. The most widespread operative technique today is bilateral sequential LTX [70]. In rare circumstances, where there is a retracted and destroyed lung in one of the thoracic cavities and a hyperdistended lung on the other side accompanied by a fixed shift of the mediastinum, a single-lung transplant combined with a pneumonectomy of the destroyed lung or combined heart-lung transplantation can be indicated.

#### 4.2.3. Operative Technique

The most common approach for CF recipients is a bilateral anterolateral thoracotomy that provides the best exposure to both lungs and the heart. Transverse sternotomy is only performed if exposure warrants it. Positioning of the patient is supine with abducted arms and the chest elevated by inflatable cushions.

During pneumonectomy, in case of a previous pleurodesis or dense pleural adhesions, special attention has to be paid to avoid phrenic and left recurrent laryngeal nerve injury while mobilizing the lungs (see below). Pneumonectomy is started with stapling of the pulmonary vessels. The bronchus is prepared centrally and cut with a scalpel, and the lung is removed from the chest cavity. Denudation of the recipient bronchus beyond the level of the planned anastomosis should be avoided by any means to prevent ischemia of the bronchial anastomosis (see below); however, bulky or calcified nodes at the recipient hilum can sometimes be a challenge during dissection. Any excessive lymphadenectomy should usually be avoided to prevent significant mediastinal bleeding, though it may sometimes be indicated to reduce infection load in recipients infected with NTM [71].

Prior to the implantation of the donor lung, it is crucial to achieve accurate hemostasis, since parts of the chest cavity are difficult to access once the lung has been implanted. In particular the posterior mediastinum can be a source of significant bleeding in CF recipients.

Donor lung preparation is usually performed immediately prior to implantation. The bronchus is shortened with only one cartilage ring remaining after the separation of the upper lobe bronchus. The peribronchial tissue has to be preserved and denudation of the bronchus has to be avoided under all circumstances to prevent bronchial healing complications (see below) [72].

#### 4.2.4. Size-Reduced LTX

Given that many CF recipients are still smaller than the average population, the waiting time until an appropriate donor lung can be identified may become unacceptably long. Therefore, several techniques have been developed to accept oversized donor organs for urgent paediatric or small adult recipients [73]. Available data show no adverse effect of pneumoreduction on survival and posttransplant spirometry, allowing safe use of larger donors in small CF recipients [74, 75].

In case of a moderate size mismatch, nonanatomical segmental resections are a straightforward technique to reduce the size of the donor lung. The middle lobe and the lingula are the preferred target areas for these resections, which are performed immediately prior to the implantation or after the lungs are implanted and perfused.

In case of a more significant size discrepancy, lobar transplantation is another option. Some specialised centres perform living-related transplantation of (usually the lower) lung lobes from two healthy relatives/donors. Living-related lobar transplantations are exceptional procedures [76]. Split lung transplantation is performed by subdividing the parenchyma of a left donor lung and replacing the right recipient lung by the donor's upper lobe with inverse anastomosis of the bronchus (pars cartilaginea to pars membranacea). The recipient's left lung is replaced by the donor's lower lobe [73].

#### 4.2.5. Extracorporeal Support

The routine use of intraoperative ECLS in bilateral LTX is still controversial. For many CF patients, extracorporeal support becomes necessary during surgery, though. This is often due to insufficient oxygenation on single-lung ventilation or hemodynamic instability, partly because of the necessity of retracting the heart to facilitate the exposure in the rather small chest cavities of these patients, but especially in patients with secondary pulmonary arterial hypertension. ECLS facilitates intraoperative cardiocirculatory and respiratory stability. They also protect the first implanted lung, which is otherwise exposed to the entire cardiac output during the transplantation of the second lung, from the consequences of uncontrolled reperfusion, which might result in reperfusion oedema and primary graft dysfunction (PGD) [62].

The most widespread intraoperative support device is cardiopulmonary bypass (CPB). However, an increasing number of institutions use venoarterial ECMO with heparin-coated cannulas instead of CPB, which avoids full heparinisation and therefore leads to reduced blood loss. This approach can be especially recommended for CF patients, who very often present with dense pleural adhesions and a resulting high risk of pleural bleeding. Intraoperative extracorporeal support can be provided either by central or peripheral cannulation over the femoral vessels [77].

### 4.3. Surgical Complications

The surgical complications occurring in LTX may be evident peri- and postoperatively up to several months after surgery. Early complications include hemorrhage, reperfusion injury, vascular obstructions, nerve lesions, and wound infections. Later complications are mostly bronchial suture healing defects.

#### 4.3.1. Bleeding

Hemorrhages after LTX are the most common complication. CF recipients in particular tend to develop more serious bleedings in the peri- and postoperative period due to pleural adhesions. If chest surgeries or pleurodeses were performed prior to LTX, the surgical risk is greatly increased [78]. It is not unusual that patients—after a successful LTX—have to return to the operating room in the immediate postoperative period for clot removal and cleansing of the pleural spaces.

#### 4.3.2. Reperfusion Injury

Reperfusion of the donor lung may lead to reperfusion injury and reperfusion edema, which may result in PGD. Clinically, PGD occurs during the first 24 to 72 hours after LTX and mimics classical acute respiratory distress syndrome (ARDS). The severity of PGD is classified following ISHLT criteria [79]. Treatment is similar to ARDS. Histology usually confirms diffuse alveolar damage. Mortality correlates with the severity of PGD and is one of the main causes of death in the early postoperative period of LTX [80]. Furthermore, PGD is also a hazard for bronchiolitis obliterans syndrome and chronic graft rejection [81].

#### 4.3.3. Vascular Complications

Surgical vascular complications may occur at the suture sites with secondary obstruction or thrombosis of the vessel. Severe and life-threatening consequences of angio-occlusions culminate in total graft failure or in a fulminant infarction. Immediate surgical repair should always be the therapeutic intervention, that is, complete thrombectomy [82, 83]. After LTX, there is also an additional increased risk of deep vein thrombosis and pulmonary artery embolism [84].

#### 4.3.4. Nerve Lesions

Phrenic nerve palsy is an infrequent, but potentially serious complication of LTX. Phrenic nerve injury may result from traction during lung mobilisation, traumatic dissection, thermal injury by electrocautery, or local topical hypothermia. If bilateral, phrenic nerve palsy may require prolonged ventilatory support either by mechanical ventilation via a tracheostomy or by noninvasive ventilation. It may recover over time [85, 86]. Recurrent laryngeal nerve palsy may also occur and lead to vocal cord paralysis.

#### 4.3.5. Airway Complications

The correct healing of the bronchial sutures is a major challenge after LTX, as the bronchial artery circulation is not reestablished and these sutures are, thus, prone to ischemia. The healing of the bronchial anastomoses is typically classified according to Whitson et al. [80]. Bronchial anastomotic complications have been significantly reduced by improved surgical techniques in recent years and in most centres the reported prevalence is <5% by now; however, wide variations are seen as there is no standardised approach to reporting these complications [87]. Bronchial anastomotic complications include necrosis, stenosis, malacia, and dehiscence [88]. Many cases can be managed conservatively; those which require treatment can mostly be solved by interventional endoscopic procedures (e.g., high-frequency diathermia, laser therapy, cryotherapy, balloon bronchoplasty, endobronchial brachytherapy, and stent implantation) [89]. Repeated surgical measures such as resurgery of anastomosis, sleeve surgery, and retransplantation are only performed in exceptional cases.

Probably because of preservation issues some patients develop bronchial complications at sites distinct of the bronchial anastomoses, such as, for example, the vanishing bronchus intermedius syndrome [90].

#### 4.3.6. Wound Infections

Due to induction therapy and consecutive immune suppression, wound infections after LTX are frequent complications. However, serious complications that require surgical (re)intervention are rather rare (<1%) [91]. A high index of suspicion must be maintained as these immunosuppressed patients do not readily develop fever or full-blown symptoms. Persisting complaints of thoracic pain warrant evaluation for wound infection, pleural and/or mediastinal infection as during dissection the thoracic cavity may be contaminated.

### 4.4. Perioperative Nutritional Management

In the immediate postoperative period, maintenance of adequate nutritional intake is a priority. Energy and protein requirements are based on recommendations for general surgical and other types of transplant patients as currently there is no specific data for CF [92]. Oral diet can usually resume on postoperative day (POD) 1 or 2 if the patient has been extubated swiftly. Oral intake should be increased as tolerated with provision of adequate pancreatic enzyme replacement therapy (PERT) [46, 93]. Appetite generally improves as breathing becomes easier, bowel function normalises, and mobility improves. However, anorexia, changes in taste, nausea, vomiting, diarrhoea, or constipation may limit oral intake [46]. These may be due to medical conditions, but a number of the medications used to prevent or treat rejection and infection, including antibiotics and antifungal drugs, can also cause nausea, vomiting, diarrhoea, and changes in taste and affect dietary intake. This is especially observed during the early postoperative period when the patients receive very complex medication regimens. Supplementary enteral feeding should be initiated if oral intake is insufficient if the patient is unable to commence an oral diet by POD 3-4 or if longer-term (non)invasive ventilation is required. Patients, equipped with a gastrostomy tube, can usually reduce the use of enteral feed as oral intake increases and nutritional status becomes adequate. At a distance from surgery removal of the gastrostomy tube can be discussed between the patient, CF team, and LTX team and assessed on an individual basis. Ideally, the removal should only occur when a BMI >19 kg/m^2^ has been achieved and maintained for 3–6 months without supplementary nutrition [94].

Following LTX, routine monitoring of fat soluble vitamin levels is recommended, as supplements may have to be reduced or discontinued. Hypervitaminosis A and E have been reported [95, 96] even after supplementation ceased. The etiology of this is unclear but may be due to altered absorption, drug interactions, impaired retinol interactions, or increased hepatic synthesis of retinol-binding protein.

Immunosuppression increases the risk of new onset secondary diabetes, especially in the immediate postoperative period or during treatment of acute rejection (AR) with high-dose steroids. Special attention must be paid to glucose controls at these times, especially in patients with no previous history of diabetes and who may not easily recognise symptoms associated with hyperglycaemia. The immunosuppressive regimen may also decompensate already existing CF-related diabetes (CFRD) [93, 97, 98]. Insulin is usually required along with tailored nutritional management to optimise blood glucose control.

Gastrointestinal complications are common post-LTX. In the early postoperative period, constipation and distal intestinal obstruction syndrome (DIOS) are common, particularly among patients with a history of DIOS, previous major abdominal surgery, or meconium ileus in infancy [99, 100]. Since DIOS is associated with severe complications such as perforation and sepsis, some centers recommend pre-op bowel preparation in all CF patients. Counselling on adequate fluid and dietary fiber intake should be provided and bowel movements should occur within the first 48–72 hours after surgery otherwise laxatives/enemas should be administered.

*Clostridium difficile* colonisation or recurrent infection is common in older CF patients and diarrhoea posttransplant should immediately trigger stool cultures and empirical treatment until stool culture results become available as* Clostridium* colitis in the setting of immunosuppression may be devastating.

The risk of osteoporosis, which is generally higher as a result of the underlying disease, is additionally increased post-LTX. Pathogenesis is multifactorial, with long-term use of corticosteroids and the calcineurin inhibitors (CNIs) (tacrolimus or cyclosporine A) as contributing factors [4]. Adequate calcium and vitamin D intake should be encouraged along with weight-bearing exercise and antibone resorptive medication as required.

## 5. Postoperative Care

### 5.1. Long-Term Follow-Up after LTX

After LTX problems arising from the genetic disorder continue to require CF specialist input, but specific posttransplant aspects have to be dealt with as well. Sometimes the CF-specific topics are predominant; at other times the posttransplant management issues are more acute. CF LTX recipients should ideally be followed in an institution where CF and LTX centre are closely cooperating and located in near vicinity in order to receive the best possible support with respect to both conditions. In most situations the transplant team will take the lead and the CF team will support this management. In real life, though, many CF patients have to travel a long way for LTX and consequently for posttransplant care are more frequently seen in their CF centres or by their general practitioners (GP). Irrespective of the model of shared care, there has to be close contact between all actors involved in post-LTX care and management. Accurate agreements about the tasks, functions of control, and coordination to be taken over by either the LTX centre or the CF centre, and/or the GP are required in the long run [101]. The patient's GP can become an important actor as he is often the first contact in case of problems and, thus, should always be informed about the patient's current status. He may also assume the task of reviewing lab tests and drug levels. However, the role of the GP in specific posttransplant care varies between countries, even in Europe.

All team members involved in post-TX CF care share the responsibility to detect signs of patient deterioration and post-TX complications as early as possible (e.g., malaise, fever, sputum production, dyspnea, decrease in lung function, diarrhoea, etc.).

As a general rule, any new respiratory symptom or sign in the immunosuppressed CF LTX recipients requires immediate attention and contact with the transplant centre. For a CF patient, who has lived with a limited lung function for years and who has had a successful LTX, this might not make immediate sense; however, after a successful LTX it is expected that the patient is free of respiratory symptoms and furthermore the immunosuppression may partially mask symptoms/signs so that even minor respiratory symptoms/signs might point to a serious problem.

New digestive problems, especially vomiting and diarrhoea, may compromise the absorption of immunosuppressive medications and, thus, expose the patient to a risk of AR and/or have a negative impact on renal function. As a consequence this type of symptoms should be taken seriously as well.

Certain viral infections, such as CMV, EBV, and varicella, can be life-threatening for transplanted CF patients [102].

Apart from LTX-related complications, CF LTX recipients are at risk for disease-specific complications that may have a significant impact on their long-term outcome. The risk of developing CFRD is markedly increased [97, 103, 104].

Considerable evidence demonstrates a relationship between gastroesophageal reflux disease (GERD) and bronchiolitis obliterans syndrome (BOS) [105].

Markers of aspiration-induced injury may be studied in BAL or induced sputum. Acid neutralisation by administering proton pump inhibitors seems to be insufficient to protect against aspiration injury [106]. In contrast, surgical therapy by fundoplication is associated with decreased levels of markers of aspiration in BAL as well as a reduced incidence of BOS or improvements in lung function in patients already diagnosed with BOS [107]. CF LTX recipients have an even significantly higher prevalence (90%) of GERD when compared to other LTX recipients suggesting that CF patients in particular should be routinely screened for GERD after transplantation to identify those who may benefit from antireflux surgery [108].

The risk of osteoporosis is further increased after LTX due to the long-term administration of corticosteroids and CNIs [4]. Close follow-up on the bacterial spectrum in the airways is indispensable as chronic infections with CF-specific pathogens can persist in the upper airways and reinfect the transplanted lung.

### 5.2. Immunosuppression

LTX recipients are usually on a triple immunosuppressive therapy combining a CNI (tacrolimus (tac) or cyclosporine A (CsA)), an antiproliferative agent (mycophenolate mofetil (MMF), mycophenolate sodium (MPS), or azathioprine (aza)), and glucocorticoids (Table 3). The immunosuppressive therapy is usually steered by the LTX centre, but monitoring is usually provided by the GP and the CF centre as well. The target levels of immunosuppressive drugs depend on the time elapsed since LTX and associated comorbidities (Table 4). An individual drug target level for each patient should be determined in short intervals and communicated to each care team member (LTX centre, GP and CF centre). Decisions of the LTX centre to wean patients down to a low steroid dose will be based on considerations of individual parameters including acute rejection (AR) episodes, the degree of renal dysfunction, and side effects.

### 5.3. Acute Rejection

The incidence of AR of the transplanted lung is high for several reasons, the most important being that there is no possibility for HLA matching. As a consequence most LTX recipients receive completely mismatched grafts [109].

Data from the ISHLT Registry indicate that approximately 35% of patients experience at least one AR episode during the first year after being discharged from the hospital [110, 111]. Apart from the obvious immunological risk factors, any inflammatory/infectious damage to the lung allograft probably increases the risk for AR. For example, viral infections—such as community-acquired respiratory viral infection or CMV infection—have been associated with an increased risk of AR [112].

The clinical picture of AR is unspecific. The complete picture of dyspnea, fever, pleuritic pain, and effusion is only observed in the immediate postoperative period. A drop of paO_2_ or FEV_1_ may provide hints. The chest CT scan may show air trapping, septal thickening, ground glass densities, bronchial dilatation, bronchial wall thickening, and centrilobular opacities, but these findings are altogether nonspecific and, most importantly, are unable to differentiate AR from infection [113].

The gold standard for the diagnosis of AR is lung histology based on transbronchial biopsies (TBBs). TBBs are assessed for acute cellular vascular and bronchiolar rejection. The severity of these processes is graded according to the ISHLT classification [100]. The A grades define vascular rejection according to the severity of perivascular and interstitial mononuclear infiltrates (A0 to A4). The B grades stage synchronous bronchiolar rejection according to the severity of  lymphocytic infiltration within the bronchiolar walls (B0 to B2R) [114]. Histological interpretation of TBBs is not always straightforward; there may be considerable interexaminer variability.

The vast majority of LTX centres will give treatment for AR graded ≥A2. Opinions differ regarding the indication for treatment in case of A1 rejection. Standard therapy consists of intravenous high-dose pulse steroid therapy (e.g., 500–1000 mg methylprednisolone daily for 3 days). Other options, mainly for recurrent or persistent AR, include antithymocyte globulin (ATG), alemtuzumab (anti-CD52), extracorporeal photopheresis, and anti-IL2 receptor antagonists.

While the existence of antibody-mediated rejection (AMR) after LTX is at present acknowledged, its diagnosis carries many pitfalls. When a diagnosis of AMR can be established, specific treatments such as high-dose steroids combined to plasmapheresis and intravenous immunoglobulins and, possibly, rituximab, bortezomib, or eculizumab may be considered though not evidence based.

### 5.4. Chronic Lung Allograft Dysfunction (CLAD)

Chronic lung allograft dysfunction (CLAD), especially bronchiolitis obliterans (BO), remains the major medium- and long-term problem in LTX and the leading cause of death.

BO is characterised clinically by progressive shortness of breath accompanied by a progressive and irreversible obstructive spirometric defect. The histological hallmark of this entity is obliteration of terminal bronchioles and evidence of aberrant remodeling in the airway epithelium, vasculature, stroma, and lymphoid system. BO is, initially at least, a heterogeneous process and histological diagnosis is difficult as TBBs are prone to sampling errors [115].

For this reason, a functional corollary, bronchiolitis obliterans syndrome (BOS), has been devised by the ISHLT. After LTX, the 2 best measurements of vital capacity (VC), FEV_1_ and FEF_25–75_ (obtained at least 3 weeks apart), are computed and averaged on a regular basis. At each consultation the patient's lung function parameters are compared to these best values. A chronic decline in lung function with an obstructive pattern and without any identifiable cause (such as AR, bronchial anastomotic complications, bronchopulmonary infections, phrenic nerve palsy, and pleural effusions) is, then, graded according to the ISHLT BOS classification. There are 5 stages: BOS 0 (no decline in lung function), BOS 0-p (potential BOS), and BOS 1–3 [116].

The clinical presentation of BO/BOS is very heterogeneous. The type of presentation, the time from transplantation to onset, and the rate of progression are all very variable. BO/BOS may present as an acute illness and imitate a respiratory infection, but very often it is at first an asymptomatic process revealed by an insidious decline in lung function. In a minority of patients BO/BOS may develop within 2 years of LTX (early-onset BOS); however, the vast majority of affected patients develop the complication at a later time-point. The rate of decline in lung function is also very variable: in some patients the decline is very rapid, in others a slow decline over years is observed. Older reports indicate that survival 5 years after LTX is 20–40% lower in patients, who had a diagnosis of BO/BOS compared to patients free from BO/BOS [117]. However, these data have potentially evolved and series, which have not differentiated BO/BOS from other conditions such as, for example, RAS (see below), probably overestimate BO/BOS mortality rates. Of note, however, early-onset BO/BOS (within 2 years) shows a worse prognosis than does late-onset BO/BOS.

Known risk factors for BO/BOS are recurrent episodes of AR, the development of anti-HLA antibodies, but also bacterial/fungal colonisation of the graft (*P. aeruginosa *recolonisation of the lung transplant is considered an independent risk factor for the development of CLAD [118]), community-acquired respiratory viral infection, CMV pneumonitis and GERD.

Prevention of BO/BOS includes addressing all known risk factors. This is the reason why at least 50% of LTX programs perform surveillance TBBs during at least the 1st year after surgery. The development of donor-specific HLA antibodies should probably also be followed prospectively, even if at present there would be no specific therapy protocol in patients becoming antibody positive.

There is no current effective therapy for BO/BOS. Once the diagnosis is established, a change of immunosuppressive therapy is often operated, most frequently to no avail. However, overimmunosuppression should be avoided in order not to trigger infectious events. A 3–6-month course of azithromycin is justified. GERD should be reevaluated and treated aggressively. All infectious episodes should be treated promptly. General supportive measures such as maintaining a correct nutritional status are important. In some circumstances, photopheresis or total lymphoid irradiation is proposed and able to stabilize lung function. In selected patients redo-LTX can be carried out.

In addition to representing a major obstacle to long-term survival, BOS also causes significant morbidity and loss of health-related QoL [119].

Historically, it has been believed that BO/BOS is the equivalent of “chronic rejection.” However, the understanding of chronic lung allograft dysfunction is continuously evolving and the term “chronic rejection” is currently intentionally avoided by many LTX specialists for several reasons (although “chronic rejection” is still an expression used by many patients and caregivers) [120]. First of all, it has become clear that alloimmune, nonalloimmune, and even autoimmune factors contribute to the development of BO/BOS; thus, BO/BOS might be the final expression of ANY aggression to the lung allograft. Secondly, there are potentially several subtypes of BO/BOS. A subpopulation of BO/BOS patients show airway neutrophilia and seem to respond relatively well to azithromycin (neutrophilic reversible airways disease). Another subpopulation of BO/BOS patients do not present with airway neutrophilia, tend to show mainly airway fibrosis with less inflammation, and are believed to be unresponsive to azithromycin. Finally, recently, it has been recognised that there are still other types of lung disorders, for example, the recently described restrictive allograft syndrome (RAS), which contribute to the respiratory morbidity and mortality after LTX [121]. RAS is characterised by restrictive ventilatory defect, infiltrates on lung CT, and peripheral foci of inflammation and fibrosis. In general, RAS shows poorer survival than BO/BOS. For all these reasons, the term chronic lung allograft dysfunction (CLAD) has been coined in order to include all these entities.

### 5.5. Infections in the Context of Immune Suppression

LTX recipients have a higher risk of infectious complications than recipients of other solid organs because of the constant exposure of the lungs to the environment, altered cough mechanism, bronchial circulation, lymphatic drainage, and the intensity of immunosuppression, which induces a state of persistent T- and B-cell dysfunction.

Infections (particularly pulmonary infections) are a frequent complication after LTX and constitute the major cause of patient morbidity and mortality [122]. Respiratory tract infections are the most common type of infection after LTX, with bacterial pneumonias being the leading cause in this category. CMV is the second most common infection followed by fungal and mycobacterial infections.

A timetable of infections—dividing the post-LTX era into 3 periods—can be established (Table 5). This categorisation is not absolute and infections may be distributed among alternative periods, particularly depending on underlying prophylactic and immune suppressive regimes [123].

*In the early period* (1st postoperative month) nosocomial infections due to bacteria and yeasts that might be observed in other surgical patients and infections due to classical CF pathogens are most frequent.

CF-specific pathogens persist in the paranasal sinuses and upper airways and may cause infectious episodes. As stated above many LTX centres refute CF patients harbouring Bcc, notably* B. cenocepacia* and* B. gladioli* because these pathogens are prone to cause immediate invasive disease with pneumonia, empyema, septicaemia, and death. Favourable long-term results in* B. cenocepacia*-positive patients are only reported in individual cases [124].* B. gladioli*, moreover, may cause skin or soft tissue infections (i.e., sternum, chest wall, and mediastinum) as well as sepsis [124, 125]. The survival for* B. gladioli-*positive patients after LTX is significantly lower than for patients with or without Bcc (with the exception of* B. cenocepacia*) [32, 126]. The reported incidence of NTM infection post-LTX is variable. Pulmonary infiltrations and/or infections of skin and soft tissue (e.g., sternal abscess, mediastinitis) predominate.

CF LTX recipients may suffer from nosocomial infection, including respiratory tract, urinary tract, skin and soft tissue, and catheter infections, or septic courses during hospitalisation. Respiratory tract and catheter-related infections are the major cause for bacteremia or sepsis. The immune suppression (induction therapy), “medical devices,” and the administration of broad-spectrum antibiotics (typically selected to cover a patient's individual spectrum of CF pathogens and their resistance phenotypes prior to transplantation) are major risk factors. Thus, the relevant pathogens comprise frequent nosocomial Gram-positive and Gram-negative bacteria such as* S. aureus* (MRSA/MSSA), Enterococci, Enterobacteriaceae (e.g.,* E. coli*,* Klebsiella* spp.,* Enterobacter* spp., etc.),* P. aeruginosa, Acinetobacter* spp., and mainly* Candida* spp. [126–129].

*In the middle period* (2–6 months after surgery) immunosuppression is maximal and this may in particular trigger reactivation of latent pathogens that were present in the lung donor or in the LTX recipient before surgery. Consequently, infections due to immunomodulating viruses (CMV, EBV, human herpesvirus 6, and hepatitis B and C) and opportunistic infections (by pathogens such as* Aspergillus* spp.,* Pneumocystis jirovecii,* and* Listeria monocytogenes*) predominate.

Cytomegalovirus (CMV) belongs to the herpes virus family and, after bacterial pneumonia, constitutes the second most common cause of infection in LTX recipients [130].

The risk of CMV infection is related to the recipient's pretransplant serological status, degree of immunosuppression, and type of antiviral prophylaxis during the early transplant period.

While short-term mortality in acute CMV infection has been reduced by CMV-killing virustatic agents, the indirect immunological effects of CMV infection predispose to CLAD [131].

CMV infection must be differentiated from CMV disease. CMV infection is characterised by an asymptomatic state of virus replication on clinical chemistry, whereas CMV disease signifies virus replication, clinical symptoms (e.g., fever, myalgias, and arthralgias), and tissue-invasive end organ involvement. After LTX, CMV pneumonitis is the predominant manifestation of CMV disease, but CMV hepatitis, gastroenteritis, or colitis is also encountered. CMV retinitis is less frequent than in AIDS patients [132].

Serologic tests (CMV antigen pp65), quantitative and qualitative PCR examination (CMV-DNA-PCR) of blood and BAL fluids or tissue samples [133], are used to establish the diagnosis. Histopathology is the gold standard of diagnosis of CMV disease. Prophylactic or preemptive treatment of CMV is recommended between 3 and 12 months after LTX depending on donor-recipient matching, CMV-negative recipients of CMV-positive donor lungs being at the highest risk of developing disease.

Due to a high risk of CLAD, it is advisable to treat LTX patients already when diagnosed with asymptomatic CMV viremia [133].

The spectrum of* Aspergillus*-related infections ranges from simple colonisation to invasive tracheobronchitis to invasive pulmonary or disseminated aspergillosis [134].

Invasive tracheobronchitis is particularly dangerous in the early postoperative period when it may affect the bronchial suture, compromising its healing with—sometimes—disastrous effects such as fistula formation between bronchus and adjacent pulmonary artery [135]. Invasive pulmonary and disseminated forms are seen mostly later on [136, 137]. Risk factors for invasive aspergillosis include colonisation prior to LTX, extensive ischemia and necrosis of the bronchial anastomoses, CMV infection, high-dose steroid therapy, and increased maintenance immunosuppression in the context of CLAD.

Preventive management is carried out up to six months after LTX or at times of increased immunosuppression; there are no standardised protocols, but individually targeted approaches have been described [138].

Before the discovery of the novel triazole-antimycotics (e.g., voriconazole, posaconazole) invasive pulmonary or disseminated aspergillosis in a LTX recipient was uniformly fatal; these drugs are nowadays the treatment of choice and allow for a better prognostic outlook [139]. When prescribing these drugs particular attention should be paid for interactions with CNIs (CNI metabolism is inhibited by azoles and, thus, blood levels increase significantly) and other drugs metabolised via the CYP3A4 enzyme cascade. Echinocandins or lipid formulations of amphotericin B are potential, but less effective, substitutes [137].

All solid organ transplant recipients are at a high risk for* Pneumocystis jirovecii* (Pj) pneumonia, especially in the first posttransplant year. This pathogen may lead to a life-threatening interstitial pneumonia. Continuous prophylactic administration of trimethoprim/sulfamethoxazole on 3 days of the week has very significantly reduced the incidence of Pj pneumonia after LTX, which nowadays is very rare. Pneumocystis and/or trophozoites are visualised in BAL by Grocott's or Giemsa's staining; PCR-based methods are also available to ascertain the diagnosis of Pj pneumonia. High-dose trimethoprim/sulfamethoxazole is the treatment of choice; concomitant steroid therapy is required if the patient is hypoxemic [140]. Side effects are common, particularly at the high doses of trimethoprim/sulfamethoxazole used in therapy (severe rash, associated with fever, cytopenia, and deterioration of kidney function). Alternatives, but less effective, are nebulised pentamidine, oral dapsone, or atovaquone suspension.

*C. difficile* is isolated from stool specimens in 30–50% of CF patients, considerably more often than in other patient groups. In the first year after LTX, CF patients are 2-3 times more susceptible to* C. difficile*-associated diarrhoea and colitis than non-CF patients [141]. Severe colitis is a rare but classical cause of death in CF LTX recipients [142, 143].* C. difficile* infection, however, may establish also after years mostly in the context of intensified immunosuppression due to graft rejection [144].

#### 5.5.1. Respiratory Syncytial Virus Infection

RSV infection after LTX has prevalence of up to 20% and, along with acute morbidity, is an independent risk factor for the development of CLAD. Diagnosis is made by detection of virus replication (RSV-DNA-PCR) in nasopharyngeal specimens. Treatment is primarily carried out with ribavirin (if available) or an increased dose of steroids [145].

Onset in the late period is mainly associated with increased immune suppression in the context of CLAD.

### 5.6. Malignancies and Lymphoproliferative Diseases

Solid organ TX, independent of the underlying disease, increases the risk of malignancy by three- to four-fold. After LTX, the Registry of the ISHLT reports that 28% of ten-year survivors developed a malignant disease [146]. The risk of malignancy increases with time. In CF LTX recipients the risk of cancer is about six times higher than in the general population, with a substantial incidence of tumours involving the gastrointestinal tract (21-fold), skin and lymphoproliferative diseases (44-fold) [147]. There are no specific guidelines for the prevention of malignant disease after LTX. It is recommended to follow at least the guidelines for the general population. Additionally, consistent application of sunscreen, avoidance of overexposure to sunlight, and regular dermatological checks are advised to prevent skin tumours. Regular screening colonoscopies, probably to be started at an earlier age than in the general population, may identify premalignant colonic lesions and prevent progression to malignancy [148].

#### 5.6.1. Posttransplantation Lymphoproliferative Disease (PTLD)

PTLD denotes various lymphoproliferative diseases (including lymphomas) occurring after TX and affecting 2–8% of the adult transplant recipients. PTLD occurs typically in the context of Epstein-Barr virus (EBV) infection or reactivation, which leads to immortalisation of EBV-infected B cells. Immunosuppression after TX inhibits T cell-mediated control of these cells leading to lymphoproliferation.

The risk for PTLD is particularly high in EBV-seronegative recipients of EBV sero-positive donor lungs (~30% compared to <5% in sero-positive LTX recipients), which also explains the even higher risk for transplanted children. Other risk factors include the type of induction therapy and the intensity of immunosuppression. PTLD can be asymptomatic in the early stages and symptoms may remain nonspecific thereafter. Imaging studies are performed according to the patients' complaints, definite diagnosis is made by histology, and the disease is categorised according to WHO recommendations [149].

Clinically, two different courses of PTLD can be distinguished. Early PTLD is observed during the first year after LTX, with frequent intrathoracic manifestations and a mostly polyclonal histology. Delayed PTLD appears later and often outside the allograft and tends to be EBV negative. Its prognosis is poorer. The most important treatment options for PTLD include reduction of immune suppression (if possible), administration of an anti-CD20 antibody (rituximab) (if the PTLD is a B cell disease), and/or chemotherapy [150]. Rescue therapies consist of autologous/allogenic stem cell transplantations.

## 6. Other Topics

### 6.1. Physiotherapy Intervention

Physiotherapy before and following LTX includes respiratory management, physical and functional rehabilitation, and education. Short-term and long-term goals need to be set.

The goal of intensified physiotherapy prior to LTX is to maintain the waitlisted patient in a clinically optimal condition for surgery. Apart from improving chest mobility and airway clearance, an individually tailored exercise program to improve functional capacity is usually started. Such a program may include aerobic conditioning as well as exercises to increase strength, flexibility, and/or balance/coordination. After LTX the main goal is to lessen chest pain and discomfort caused by the clamshell incision, improve breathing and cough while improving physical fitness as quickly as possible to avoid immobilisation, and to allow the patient to perform everyday activities [151].

#### 6.1.1. Types of Physical Training

Physical training is defined as participation in a program of regular vigorous activity designed to improve physical performance or cardiovascular function or muscle strength or any combination of these three [152]. In general, people with CF can benefit from a wide range of different training modalities, for example, aerobic, anaerobic, and strength-training. Short- and long-term aerobic and/or anaerobic training have been proven to result in positive effects on lung function, aerobic fitness, strength, and quality of life [153–155].

In people with severely impaired pulmonary function and muscle wasting, interval training or moderate strength training might be better tolerated than continuous aerobic exercises. Although the evidence is sparse, support by oxygen supplementation [156] and noninvasive ventilation may allow/facilitate physical training in patients with severely altered respiratory function.

Aerobic training may include cycling or walking for a prescribed length of time at a targeted intensity, or interval training. Often, aerobic training intensity is based on a standardised exercise test. Some rehab facilities use the steep ramp test originally developed for cardiac rehabilitation [157] to guide interval training. This approach has been adopted for rehab in pulmonary diseases [158] and also used successfully in CF patients before and after LTX [159].

Although the data on severely ill patients is limited, clinical experience shows that supervised strength training is feasible and safe and sometimes better tolerated than endurance type exercises. Sufficient time for recovery, that is, more than 48 hours, is required following an intense training session.

#### 6.1.2. Before TX

Standardised exercise testing is routinely used in the pretransplant assessment [160–162]. Currently, most centres employ the 6-minute walk test [161]. However, other exercise tests, including full cardiopulmonary exercise testing, are also used and may provide additional information [161, 162]. Besides providing prognostic information [160, 162], the exercise test can guide advice on exercise training and conditioning during the waiting period and during posttransplant rehabilitation.

Preoperative rehabilitation aims to achieve optimum conditioning with regard to strength, endurance, mobility in everyday life and nutritional condition, at creating a better starting point for the transplantation thereby making for a better postoperative outcome [163].

The poorer a patient's condition is, the more he/she is going to benefit from an individually adjusted training program [164]. Preoperative rehabilitation may furthermore add to the selection of appropriate candidates or to the determination of the listing point. Contraindications for LTX—which previously were gone unnoticed—may become obvious during rehab. In many cases prior to LTX, CF patients do not meet the defined criteria for rehabilitation. Cardiopulmonary performance is frequently restricted to the extent of immobility, and the patients are incapable of taking care of themselves. Pulmonary rehabilitation must be oriented along the individual limitations and capacities of the LTX candidates, and this can only be done in specialised centres. Disease-specific features have to be taken into account, for example, bacterial colonisation in CF patients.

Besides the inhalation treatment and airway clearance sessions, patients should have access to exercise training three times a week in order to ensure that they are in an optimal condition for surgery. The individualised programme needs to be regularly reviewed by an expert physiotherapist or exercise specialist and regular assessments are required to adjust the programme to possible improvements in fitness or deterioration of general health.

#### 6.1.3. After TX

Physiotherapy programs should be initiated in the ICU setting. Early mobilisation and daily physiotherapy is essential [165], taking into account the individual limitations of performance. The benefits of postoperative rehabilitation after LTX include reduced dyspnoea and subjectively an improved general condition [166]. A clinically relevant improvement of the walking distance (6-minute walk test) [167] and improved QoL after LTX [168] can be accomplished by individualised endurance and strength training.

Physiotherapy and respiratory therapy play a key role for patients with CF even after the transplantation. Aside from improving the elimination of secretions in the early postoperative period, it is essential to counteract limited ventilation due to thoracic restriction and pain related to scars/contractures. Conditioned pathologic breathing patterns must be compensated by correcting the patients' respiratory perception. This is also paramount since perception in the transplanted organ had been reduced by surgical denervation [169, 170].

After successful transplantation, physiotherapy must be continued past the period of early rehabilitation. Patients falsely overestimate their performance after LTX [166]. Continued resistance workouts are important to counterbalance the effects of long-term steroid intake.

### 6.2. Other Solid Organ Transplantations in CF (Heart, Liver, Kidney, and Pancreas)

Heart-lung transplant is a major procedure that carries a high risk of complications. It is rarely performed in CF at present due to the fact that after bilateral lung transplantation myocardial recovery from right heart failure is dramatic and obviates the need for cardiac replacement [171].

The occurrence of liver disease in CF (CFLD) is specified at 27–35% by age of 18 years [172]. Incidence is at 2.5 per 100 patient years in the first ten years of life, with a prominent decline in the second decade. 5–10% of all patients will develop multilobular cirrhosis [173]. Liver transplantation (LiTX) is the treatment of choice in advanced CFLD. The indication for this procedure in CF differs compared to other diseases with chronic hepatic failure. Extrahepatic factors have to be considered as well, for example, progressive deterioration of nutritional condition (catabolism) and particularly pulmonary factors such as deteriorated lung function and an increased number of pulmonary exacerbations. Paediatric patients will benefit from early LiTX with improved lung function, since pulmonary alterations are more likely to be reversible in them than in adults. Given that particularly in CF disease severity and urgency of transplantation cannot be defined by common scoring systems like MELD (model of end-stage liver disease) or PELD score for paediatric patients [174], CF was added to the group of “standard exceptions” (exceptional and match-MELD score) to facilitate a better allocation of donor organs and improve the chance of a timely transplantation. Indication is clear in case of progressive hepatic dysfunction, uncontrollable ascites and variceal hemorrhages, hepatopulmonary syndrome, severe malnutrition. reduced QoL, and declining lung function due to advancing decompensation. A typical contraindication is poor pulmonary function. The threshold value for LiTX alone has been set empirically rather than evidence based at FEV_1_/FVC <50% [175–177]. In any case, LiTX should be performed before lung function declines significantly. Immunosuppressive therapy does not differ here from other LiTX patients; however, CF patients show fluctuating drug levels more frequently and may require closer monitoring. CF patients develop diabetes mellitus more frequently than others after LiTX, whereas the prevalence of reduced renal function at 2 and 5 years is similar to that of LiTX recipients without CF [177]. CFLD treatment and liver transplantation have a positive effect on lung function in both children and adults; the long-term course of lung function is not truly influenced by LiTX, though, and matches that of patients without CFLD [178]. Inversely, CFLD does not constitute a contraindication to planned LTX, provided that hepatocellular function is conserved and portal hypertension is controlled [179].

In rare cases combined liver and lung transplantation has been performed, but the risk of perioperative complications is high and larger series are not available [180, 181]. In one small series the overall survival of those receiving a combined liver and lung transplantation was 70% at one and three years [181].

In rare instances a CF patient requires combined or sequential lung and kidney transplantation [182]. There are some reports on transplantations of pancreas, simultaneous liver-pancreas, or lung-pancreas [183, 184].

However, a recent meta-analysis of abdominal organ transplantation in CF concludes that pancreatic transplantation in CF is not an established procedure [185].

### 6.3. Pregnancy after Lung Transplantation

Successful pregnancy is possible after lung transplant, even among recipients with a diagnosis of cystic fibrosis [186]. However, these pregnancies are high-risk and require close maternal and fetal surveillance through coordinated care among maternal-fetal medicine specialists and transplant personnel. Little is known about how the profound immunologic changes associated with pregnancy influence tolerance or rejection of the allograft and lung recipients appear at greatest risk for poorer pregnancy outcomes. Recipients should be in general good health and there should be optimal control of comorbid conditions such as liver function and diabetes prior to conception. Graft function should be stable and rejection-free. During pregnancy, maintenance-medication regimens should be continued with vigilant monitoring for effective drug levels and drug side effects with appropriate dose adjustment. Transplant professionals have to be aware of any additional risk to the fetus from immunosuppressive medications relative to the potential improvement in maternal graft function/survival conferred by each of these agents (e.g., mycophenolic acid is associated with a 23% incidence of birth defects) [187].

With the constant advent of new developments and modifications in immunosuppressive regimens, clinicians are responsible for providing pregnancy counselling to all pre- and posttransplant recipients of childbearing age. Caregivers have to be aware of how they relay the data and appreciate how recipients will process this information in order to make an informed decision regarding parenthood after transplantation, even in the face of potential risks. Ultimately, the patient has to be given the opportunity to make the decision [188].

### 6.4. Patient Education and Learning Objectives after LTX

After lung transplantation, CF patients have to acquire a lot of new skills to manage their life with a new chronic condition: adjust to new therapy, nutrition, and care regimens; manage new medications and risks; and control new emergency situations like rejection and infection. Multidisciplinary transplant teams should offer structured patient-centred education programs, with training sessions ensuring that the patient will be able to effectively manage unpredictable situations, especially those jeopardizing the outcome of the graft. Education can be facilitated by visual aids and written booklets.

Some learning objectives are crucial and should be acquired between the time of transplant and the first return home (Table 6) [189, 190]. Detailed explanations should be provided as to the mechanism of action/absorption of immunosuppressive drugs and their anticipated side effects, the need for prophylactic anti-infectious therapy, and drug-drug and drug-food interactions. Patients with CFRD should receive ongoing diabetes self-management education, and education about the symptoms, prevention, and treatment of hypoglycemia is recommended for patients with CFRD and their care partners [191].

Other learning objectives should be addressed in the rehabilitation clinic and during the post-LTX outpatient visits, tailored to the patient situation and needs (e.g., importance of hydration and quantity of dietary salt, environmental risk factors, use of contraception, and prevention of sexually transmitted diseases, etc.) [192]. The use of knowledge questionnaires and other tools like clinical cases and scenarios facilitates the achievement of these objectives.

Finally, attention should be given to socioeconomic conditions and regular meetings with the social worker should be scheduled to help patients manage their time when they resume work. Partners and close family members or friends are of great help for transplant recipients and can be invited to attend some of the education sessions.

## Figures and Tables

**Table 1:** Preparatory tests for LTX*.

(i) Lab tests with blood group, HLA typing, and anti-HLA antibodies	
(ii) Assessment of vaccination status, booster injection if necessary	
(iii) Pulmonary function tests: body plethysmography, measurement of diffusion capacity, and standardised exercise test	
(iv) Chest CT without contrast agent, preferably not older than 6 months	
(v) Blood gas analysis at rest	
(vi) Current sputum culture	
(vii) ECG, echocardiography with evaluation of pulmonary artery pressure, and right ventricular function	
(viii) Right heart catheter if necessary	
(ix) Assessment of nutritional status	
(x) Abdominal sonography (including recording signs of portal hypertension), abdominal CT if necessary	
(xi) Gastroscopy and colonoscopy if necessary	
(xii) ENT examination, with sinus CT scan if necessary, throat and sinus swabs if necessary	
(xiii) Bone density scan	
(xiv) Gynaecological/urologic screening	
(xv) Psychological assessment	
(xvi) Dental examination	
(xvii) Presentation at ophthalmologist	
(xviii) Presentation at dermatologist	
(xix) Duplex sonography of the afferent arteries if necessary	
(xx) Peripheral closing pressure of the ankle arteries if necessary	

*Listing reflects consensus of the ECORN-CF working group. Some centres may request further investigations.

**Table:** (a) Absolute contraindications to LTX

(i) Malignant diseases in the past 2 years	
(ii) Untreatable severe dysfunction of another important organ system (heart, liver, and kidney) not amenable to surgical correction/combined TX	
(iii) Chronic, incurable extrapulmonary infection	
(iv) Severe deformations of chest and spine	
(v) Severe or symptomatic osteoporosis	
(vi) Lack of adherence to therapy	
(vii) Untreatable mental disorders combined with lack of cooperation	
(viii) Addictive disorder currently or during the past 6 months (tobacco and alcohol addiction, substance abuse)	

**Table:** (b) Relative contraindications to LTX

(i) Age > 65 years	
(ii) Critical/unstable clinical situation	
(iii) Seriously limited functional status without potential for rehabilitation	
(iv) Colonisation with *Burkholderia cenocepacia*, *Burkholderia gladioli* and *Mycobacteria abscessus *	
(v) Diseases not optimally treated (e.g., arterial hypertension, diabetes mellitus, GERD, osteoporosis, and coronary heart disease)	

**Table 3:** Typical baseline immunosuppression beyond one year after lung transplantation*.

Therapy	Dosing
Prednisone/prednisolone	5 mg daily

Mycophenolate mofetil or mycophenolic acid	500–1500 mg twice daily 360–720 mg twice daily

Cyclosporin A or tacrolimus	Targeting blood level of 150–200 ng/mL Targeting blood level 8–10 ng/mL

*Please note that patient-specific dosages have to be taken into account.

**Table 4:** Cyclosporin and Tacrolimus can be monitored by trough levels.

Posttransplant period	Cyclosporin A (ng/mL)^∗#^	Tacrolimus (ng/mL)^∗#^
0–2 weeks	300–350	10–15
3–8 weeks	250–300	10–15
2-3 month	200–250	10–15
3–6 month	190–250	10–15
6–12 month	150–200	5–10
>1 year	150–200	5–10

*Please note that reference values for drug levels are given as guidance only and may vary between centers. Patient-specific drug levels have to be taken into account.

^#^In some centres postdose levels are monitored. Specific reference values are necessary.

**Table 5:** Timetable of infections following LTX.

1st month	2nd–6th month	>6 months
Nosocomial infections* Respiratory tract infections Surgical site infections Urinary tract infections Catheter infection, sepsis	**Reactivation of latent infections + opportunistic infections**	**Community-acquired infections/pneumonia**

Related more to surgery and intensive care	Related more to immunosuppression	

CF lung pathogens: *Pseudomonas* spp. *Burkholderia cepacia* complex *B. gladioli*; NTM Other bacteria: *S. aureus* *Enterobacteriaceae* Enterococci *Acinetobacter* spp. Fungi: *Candida* spp. *(Aspergillus* spp.) Viruses: Herpes simplex virus Respiratory viruses	Viruses: Cytomegalovirus Epstein-Barr virus Herpes simplex virus Varicella Zoster virus Opportunists: *P. jirovecii* Toxoplasmosis *Aspergillus *spp. Nocardia Listeria Mycobacteria (especially NTM) CF-lung pathogens: *Pseudomonas* spp. *Burkholderia* spp.	Viruses: Epstein-Barr virus Respiratory viruses Respiratory bacteria: * S. pneumonia* *H.influenzae* *C. pneumoniae* *M. pneumoniae* and others CF lung pathogens: *Pseudomonas* spp. *Burkholderia* spp. Fungi: *Aspergillus* spp.

*C. difficile* infection**		Late-onset *C. difficile* infection

*May occur also in later periods after LTX depending on prolonged or recurrent hospitalisation and the presence of medical devices.

**Highest incidence within the first 3 up to 12 months after LTX in association with broad antimicrobial therapy and intense immunosuppression.

**Table 6:** Learning objectives for the time between LTX and first return home.

(i) Identify warning signs of a change in respiratory status (including results of spirometry/PFT)	
(ii) Respond to warning signs of a change in respiratory status	
(iii) Describe the vital nature of immunosuppressants (role, mode of action, lifelong therapy, importance of biological monitoring)	
(iv) Comply with proper handling of immunosuppressants	
(v) State the role and mode of action of other medications	
(vi) Respond to forgetting a medicine or to vomiting	
(vii) Manage the stock of drugs and equipment	
(viii) Identify food-related risks	
(ix) Prevent skin diseases in the context of immunosuppression (sun exposure)	
(x) Share their projects and activities, express their fears, desires, talk about body image, and manage stress, emotions, and so forth (life skills).	
(xi) Know the risks of travelling	
