# Erratum to “Practical Guidelines: Lung Transplantation in Patients with Cystic Fibrosis”

**DOI:** 10.1155/2015/698460

**Published:** 2015-01-28

**Authors:** T. O. Hirche, C. Knoop, H. Hebestreit, D. Shimmin, A. Solé, J. S. Elborn, H. Ellemunter, P. Aurora, M. Hogardt, T. O. F. Wagner, ECORN-CF Study Group, Thomas Becher, Scott Bell, Stefan H. Blaas, Sophie Buchberger, Lesley Carson, Isabelle Danner-Boucher, Valérie David, Karleen De Rijcke, Doris Dieninghoff, Alistair Duff, Sigitas Dumčius, Lieven Dupont, Natalie Firlei-Fleischmann, Rainald Fischer, Wolfgang Gleiber, Jens Gottlieb, Jutta Hammermann, Peter Jaksch, Barbara C. Kahl, Reem Kanaan, Marythé Kerbrat, Holger Kirsch, Christoph Lais, György Lang, Fred Lessire, Jochen G. Mainz, Araxie Matossian, Konstantin Mayer, Lisa Morrison, Annette Pfalz, Sophie Ravilly, Stefanie Rosenberger, Carsten Schwarz, Fiona Shaw, Olaf Sommerburg, Gemma Stanford, Sivagurunathan Sutharsan

**Affiliations:** ^1^Department of Pulmonary Medicine, German Clinic for Diagnostics (DKD), Wiesbaden, Germany; ^2^Department of Chest Medicine, Erasme University Hospital, Brussels, Belgium; ^3^Department of Pediatrics, University Hospital Wuerzburg, Germany; ^4^Regional Adult CF Centre, Belfast City Hospital, Belfast, UK; ^5^Lung Transplant Unit, Hospital Universitario La Fe, Valencia, Spain; ^6^Centre for Infection and Immunity, Queens University, Belfast and Adult CF Centre BHSCT, UK; ^7^Cystic Fibrosis Center, Department of Pediatrics, Medical University Innsbruck, Innsbruck, Austria; ^8^Paediatric Respiratory Medicine and Lung Transplantation, Great Ormond Street Hospital, London, UK; ^9^Institute for Medical Microbiology and Infection Control, Goethe University Hospital, Frankfurt, Germany; ^10^Department of Pneumology, Goethe University Hospital, Frankfurt, Germany; ^11^Department of Pneumology, Frankfurt University Hospital, Building 15B, Theodor-Stern-Kai 7, 60590 Frankfurt, Germany

The names of the members of the ECORN-CF study group are not listed correctly in “Practical Guidelines: Lung Transplantation in Patients with Cystic Fibrosis.” Please find below a list of the names in the correct order:

 Thomas Becher
 Scott Bell
 Stefan H. Blaas
 Sophie Buchberger
 Lesley Carson
 Isabelle Danner-Boucher
 Valérie David
 Karleen De Rijcke
 Doris Dieninghoff
 Alistair Duff
 Sigitas Dumčius
 Lieven Dupont
 Natalie Firlei-Fleischmann
 Rainald Fischer
 Wolfgang Gleiber
 Jens Gottlieb
 Jutta Hammermann
 Peter Jaksch
 Barbara C. Kahl
 Reem Kanaan
 Marythé Kerbrat
 Holger Kirsch
 Christoph Lais
 György Lang
 Fred Lessire
 Jochen G. Mainz
 Araxie Matossian
 Konstantin Mayer
 Lisa Morrison
 Annette Pfalz
 Sophie Ravilly
 Stefanie Rosenberger
 Carsten Schwarz
 Fiona Shaw
 Olaf Sommerburg
 Gemma Stanford
 Sivagurunathan Sutharsan

